# Scalable workflow for characterization of cell-cell communication in COVID-19 patients

**DOI:** 10.1371/journal.pcbi.1010495

**Published:** 2022-10-05

**Authors:** Yingxin Lin, Lipin Loo, Andy Tran, David M. Lin, Cesar Moreno, Daniel Hesselson, G. Gregory Neely, Jean Y. H. Yang

**Affiliations:** 1 Charles Perkins Centre, The University of Sydney, Sydney, Australia; 2 School of Mathematics and Statistics, The University of Sydney, Sydney, Australia; 3 Laboratory of Data Discovery for Health Limited (D^2^4H) Science Park, Hong Kong, China; 4 School of Life and Environmental Sciences, The University of Sydney, Sydney, Australia; 5 Department of Biomedical Sciences, Cornell University, Ithaca, New York, United States of America; 6 The Centenary Institute of Cancer Medicine and Cell Biology, The University of Sydney, Sydney, Australia; Harvard School of Public Health, UNITED STATES

## Abstract

COVID-19 patients display a wide range of disease severity, ranging from asymptomatic to critical symptoms with high mortality risk. Our ability to understand the interaction of SARS-CoV-2 infected cells within the lung, and of protective or dysfunctional immune responses to the virus, is critical to effectively treat these patients. Currently, our understanding of cell-cell interactions across different disease states, and how such interactions may drive pathogenic outcomes, is incomplete. Here, we developed a generalizable and scalable workflow for identifying cells that are differentially interacting across COVID-19 patients with distinct disease outcomes and use this to examine eight public single-cell RNA-seq datasets (six from peripheral blood mononuclear cells, one from bronchoalveolar lavage and one from nasopharyngeal), with a total of 211 individual samples. By characterizing the cell-cell interaction patterns across epithelial and immune cells in lung tissues for patients with varying disease severity, we illustrate diverse communication patterns across individuals, and discover heterogeneous communication patterns among moderate and severe patients. We further illustrate patterns derived from cell-cell interactions are potential signatures for discriminating between moderate and severe patients. Overall, this workflow can be generalized and scaled to combine multiple scRNA-seq datasets to uncover cell-cell interactions.

## Author summary

Despite the availability of several studies of single-cell transcriptomics profiles from different geographic locations, our knowledge of cell-cell interactions across distinct disease states and how such interactions may drive pathogenic outcomes remains limited. Motivated by the need to gain insights into health and disease and to address challenges associated with the compilation and exploration of multiple large-scale data, we developed a generalizable and scalable workflow for identifying cells that are differentially interacting across COVID-19 patients with distinct disease outcomes. Our workflow shows how to characterize cellular communication patterns for patients with varying disease severity and thus aids in the understanding of disease progression. We show the scalability and interpretability of our approach by combining around half million of cells from eight COVID-19 scRNA-seq experiments to demonstrate that individuals have heterogeneous communication patterns. Such patterns are potential signatures to discriminate between moderate and severe patients. Overall, this workflow can be generalized and scaled to combine multiple scRNA-seq datasets to uncover cell-cell interactions in complex diseases.

## Introduction

Single-cell technologies have grown in popularity as a new and powerful technique for profiling transcriptomes at the single cell level. The growing availability of single-cell RNA-seq (scRNA-seq) datasets has led to an exponential increase in the development of computational tools that can effectively use scRNA-seq data to address biological research questions [1–3]. Together, these technologies and tools have enabled ultra-high-resolution studies of cell heterogeneity, developmental dynamics, and cellular communication across diverse biological systems, which are being used to better understand the underlying mechanisms of complex diseases [4,5]. In particular, scRNA-seq has enabled modelling of cellular communication by estimating cell-type specific ligand-receptor patterns in complex tissues and relating such information to disease mechanisms such as disease progression in viral infections [6–8].

The COVID-19 pandemic caused by the SARS-CoV-2 virus has affected the global population in the last year. The majority of SARS-CoV-2 mechanistic studies have focused on the respiratory system, as SARS-CoV-2 is spread via airborne transmission [9] and Angiotensin-converting enzyme 2 (ACE2) which serves as the primary SARS-CoV-2 receptor is expressed in human airways [10–12]. It is well understood that SARS-CoV-2 infection causes a wide range of symptoms, with patients being asymptomatic, exhibiting mild symptoms, or developing severe disease with an increased risk of death [13,14]. A number of studies have suggested that the disease outcome may be determined by a combination of direct viral effects on patient tissues [15], protective antiviral immunity [16], and exaggerated antiviral or inflammatory immune responses that cause tissue damage [17,18]. However, it remains unclear why some patients have mild symptoms while others die from the illness.

In this specific context, there has been a growing collection of data and studies aimed at identifying disease progression markers by examining different tissues [19–26]. Most of these efforts have employed multiple omics technologies for an association analysis to identify differentially expressed genes, proteins or metabolites [23,24,27]. Furthermore, to define the cellular transcriptional responses involved in COVID-19 severity, single-cell RNA-seq has been performed on patient samples, including peripheral blood mononuclear cells (PBMCs) and bronchoalveolar lavage. These studies further reinforce the notion that excessive inflammation correlates with negative disease outcome [22,26]. To date, most studies focus on cell identification and cellular profiling within the cell types [28]. The current literature has not closely studied the cellular communication among immune cell types, or between epithelial and immune cell types, and how such communication affects disease progression.

Individual cells communicate with one another to modulate gene expression by neighboring cells, determine their spatial and temporal location within a tissue or organism, and transmit signals of damage or infection by external agents. Computational approaches have recently been developed to identify potential cell-cell interactions (CCIs) based on the expression of known ligand-receptor pairs in scRNA-seq data. However, current approaches are non-scalable, and unable to incorporate the increasing number and size of scRNA-seq datasets available. Such limitations are particularly evident in recent studies of scRNA-seq data from COVID-19 patients. While scRNAseq studies have generally focused on identifying peripheral blood mononuclear cells (PBMCs) and their expression profiles, the activation and subsequent response of PBMCs to infection is initiated through CCIs between lung epithelial cells and those of the immune system [29,30]. Determining how this initial interaction impacts subsequent downstream effects on PBMC activation is essential for unraveling the differential clinical response among COVID-19 patients [31]. This limitation is due, in part, to the lack of a scalable workflow for scientists to systematically harness the power of single-cell analysis to infer cell-cell interactions [7,32,33], which has the potential to inform disease mechanisms.

Here, we present a generalizable workflow and an interactive resource for exploring cell-cell interactions using a large collection of single-cell COVID-19 data sets to evaluate the molecular patterns associated with disease severity. We show how our generalizable workflow can analyze cell-cell communication networks from patients with varying disease severity and identify critical cell types and cell-cell communication channels that indicate healthy network communication. The effective integration of six different PBMC studies representing over 150 individuals with approximately half a million cells enables us to examine cellular communication between immune and epithelial cells and exemplifies the scalability of our workflow. Recognizing the importance of cell-cell communication networks within the infected lung, our workflow allows us to investigate an unexplored phenomenon in COVID-19 by studying the cellular communication between epithelial and immune cells and developing a model to discriminate disease severity.

## Results

### Generalizable workflow to identify and measure cell-cell communication in individuals

**Fig 1:**
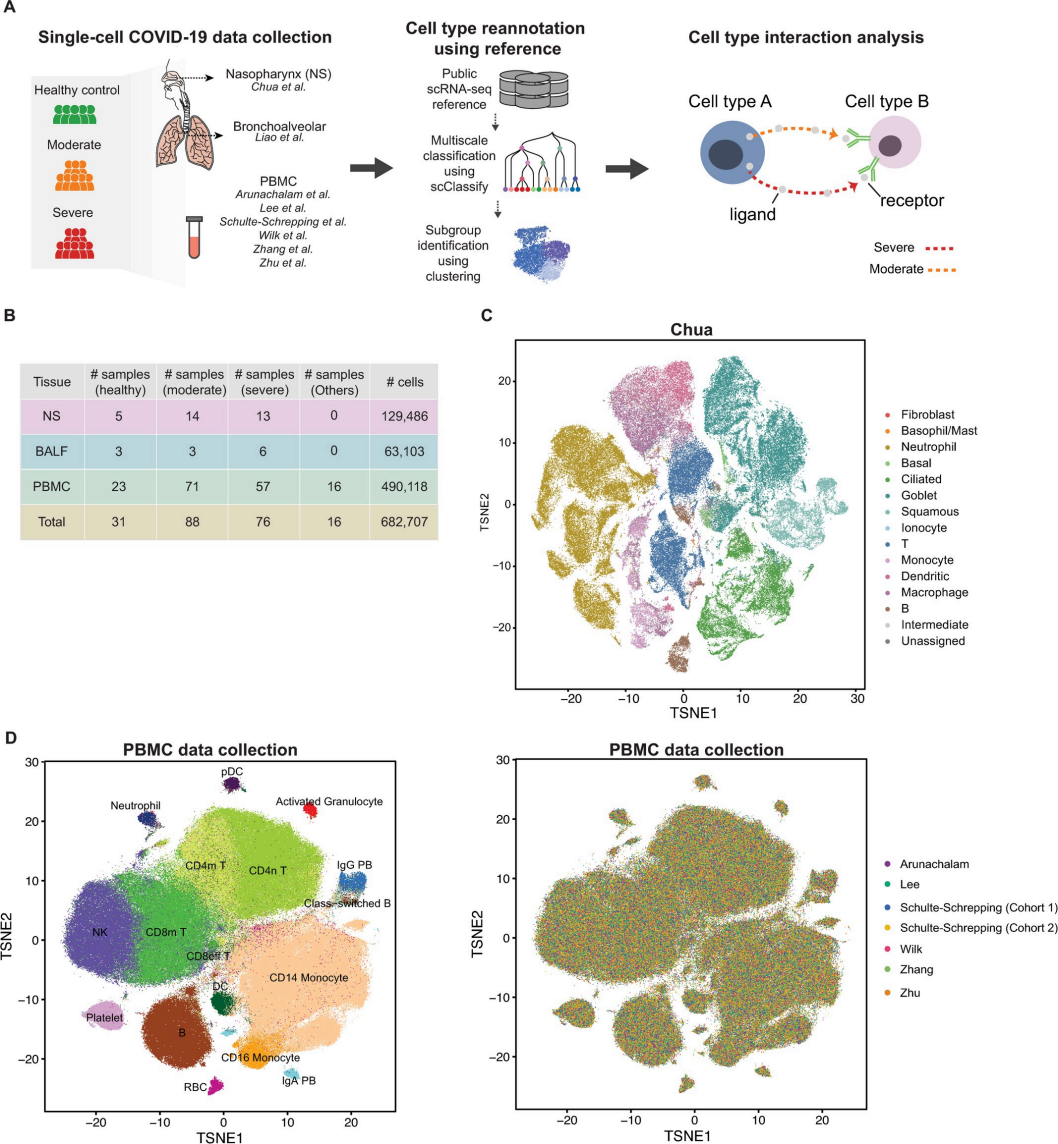
Integration and annotation of single-cell COVID-19 data. Schematic of the data analytic workflow. A. Summary of curated single-cell RNA-seq from COVID-19 studies from different tissues that are publicly available. B. tSNE plot illustrating cell types from all samples in the Chua dataset based on the reannotation using a modified version of the joint classification from scClassify built from four large reference datasets of human lungs. C. tSNE plot of the integrated matrix generated from scMerge illustrating cell types from all six PBMC datasets [21–26] curated for this study based on the reannotation using a modified version of the joint classification from scClassify built from Wilk dataset, colored by cell type (left panel) and dataset (right panel).

We develop a generalizable workflow based on statistical learning strategies that allows us to visualize, identify and characterize cell-cell interaction patterns (Fig 1A). The workflow begins with joint classification using scClassify [28] based on single or multiple reference datasets (see Material and Methods) to refine cell type annotations. The choice of reference dataset(s) depends on the availability of high quality and well annotated data for a given tissue. Next, to partition cell heterogeneity, unsupervised clustering is performed on each annotated cell type to further define subgroups of cells with the potential to identify cellular subtypes associated with different disease progression. Cluster merging [34] is used here to prevent overclustering. Finally, we calculate a cell-cell interaction score/measure for each individual COVID-19 sample between different cellular subtypes. Applying this workflow to single-cell data with multiple individuals will generate a large matrix for each individual sample with columns representing cell types and rows representing ligand-receptor pairs (Fig 1A). Each ligand-receptor pair is further grouped into different pathways to facilitate interpretation. Details of this workflow are described in the Material and Methods section.

We first examined two publicly available single-cell RNA-seq datasets from COVID-19 patients with different degrees of severity, using samples from nasopharyngeal (NS, [19,20]) or bronchoalveolar lavage fluid (BALF, Liao et al., 2020), detailed in Fig 1B. We re-annotated the cells using four healthy human lung scRNA-seq datasets [12,35,36] including 189,967 cells and 44 cell types. For the Chua dataset with 5 healthy controls, 14 moderate and 13 severe samples (S1A and S1B), scClassify is able to identify additional cell types and provide further refinement as illustrated in Fig 1C. For example, the original “outliers epithelial” cluster was refined to “ciliated cells”, and “secretory” cells to “goblet” cells. By accounting for such refinement, the new annotation recapitulates 78% of the original published analysis. The classified cell types are clearly identified by known markers (S1C), and further clustering of the Chua dataset generates 50 subclusters. Similar reannotation is applied to the Liao dataset (S2A and S2B Fig) resulting in 15 cell types and 52 subclusters.

### Cell-cell interactions are significantly different in patients with COVID-19 compared to healthy individuals

To provide insight into how these identified cell-types interact and how such cell-cell interactions (CCIs) contribute to disease severity, we calculate the CCI scores that represent the communication probabilities among all pairs of subclusters across all ligand-receptor pairs, using CellChat (see Material and Methods for details) [7]. Our group-specific CCI scores (CCI_group_) aggregate the scores across all different pathways between each major cell type pair for different disease severity groups, represented as a network graph with thicker edges indicating stronger cell-cell interaction.

Our results highlight that different patterns of cell-cell interactions occur between healthy controls and COVID-19 patients in BALF samples. We observed that most cell-cell interactions in healthy samples are between basal, ciliated, and goblet cells of the lung epithelium, with dendritic cells providing immune surveillance (S3A Fig). As disease severity increases, cell-cell interactions become dominated by interactions between the lung epithelium and proinflammatory players within the immune compartment (S3A–S3C and S4A–S4E Figs). Overall, we observe significantly less communication (fewer edges in S3A Fig) in healthy individuals compared to moderate (S3B Fig) and severe patients (S3C Fig).

### Scalable workflow to combined datasets across 6 data sets with 167 individuals

Next, we illustrate the scalability of our workflow by applying our workflow to all six PBMC datasets with 490,118 cells in total [21–26]. We unify the cell type annotation using the Wilk dataset (with 44,721 cells and 20 cell types) as a reference (Figs 1D and S5). Despite the distinct cell type compositions observed in the six studies, Fig 1D shows that cells with the same annotation were well integrated using the data integration approach scMerge [37] enabling us to examine the interaction between the various cells types.

While similar cell types exist in the six studies, a detailed look at the compositional differences across all these individuals demonstrate clear differences and these are likely the result of different sampling or cell isolation procedures (S5 Fig). Thus, cell type composition alone from single-cell experiments may not provide sufficient discriminative power to distinguish between patients with different disease severity. This, along with the varying cell-cell interaction patterns across disease groups supports the further examination of the association between cell-cell interaction with disease outcomes and progression using a workflow capable of integrating multiple datasets.

### Increased cell-cell interaction with neutrophils in severe COVID-19 patients in both PBMC and lung tissues

**Fig 2:**
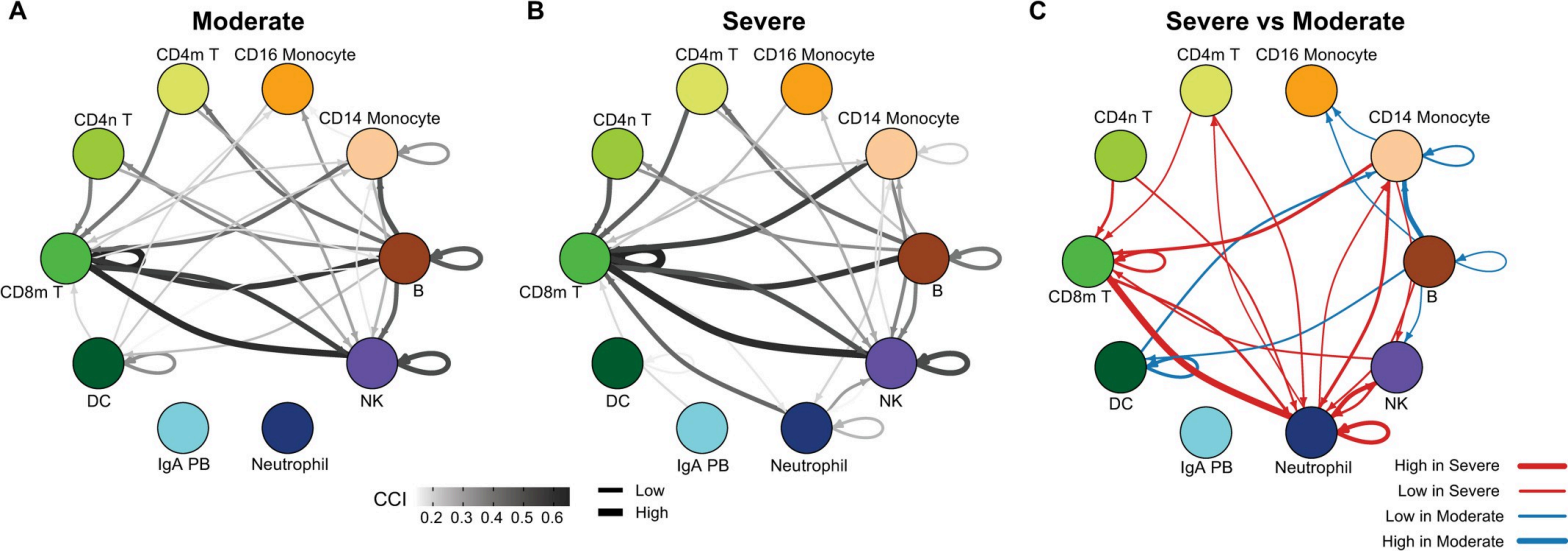
Comparison of cell-cell interactions in COVID-19 patients in PBMC of varying severities. A—B. Network representing the group specific cell-cell interaction (CCI_group_) considering different disease severity as groups in the six PBMC datasets from (A) moderate patients and (B) severe patients. The nodes represent major cell types and the edges represent aggregated tCCI interaction signals across individuals from the same group. Thicker and darker edges indicate stronger cell-cell interaction signals. Only the edges with top 10% tCCI signals are illustrated in the network. C. Network representing the difference of cell-cell interaction between severe and moderate patients. The nodes represent cell types and an edge measures the difference in cell-cell interaction. A red edge indicates an interaction higher in severe patients and a blue edge indicates an interaction higher in moderate patients.

We began by examining the combined PBMC data where the CCI scores are calculated using the scMerge corrected matrix to adjust for the dataset effect (Figs 2 and S6). Fig 2A and 2B depict two networks that represent the cell-cell interactions of PBMC data for moderate and severe patients, respectively. Each node represents a major cell type, and the edges reflect aggregated total CCI (tCCI) interaction signals where only the top 10% of tCCI signals are shown (see Material and Methods). It should be noted that in some studies [21] neutrophils are generally excluded during purification, and thus the cell type composition of neutrophils varies greatly across the six PBMC datasets (S5 Fig). Incorporating data integration in our workflow facilitates the examination of neutrophil-related cell type interaction. As expected, the network of differences reveals a considerable increase in significant neutrophil-related interaction in severe patients compared to moderate patients (Fig 2C).

Similar neutrophil-related interactions are also observed in the upper airway. In our examination of the Chua and Liao datasets S2C and S3D Figs show that the interaction is higher between monocyte/macrophage towards neutrophils in severe patients, consistent with previous findings [38]. Together, these data provide validation that our workflow can confirm known mechanisms and highlight new biology for further investigation.

### Monocyte/Macrophage and neutrophil interaction in severe patients are dominated by CXCL, IL1 and other inflammation pathways

**Fig 3:**
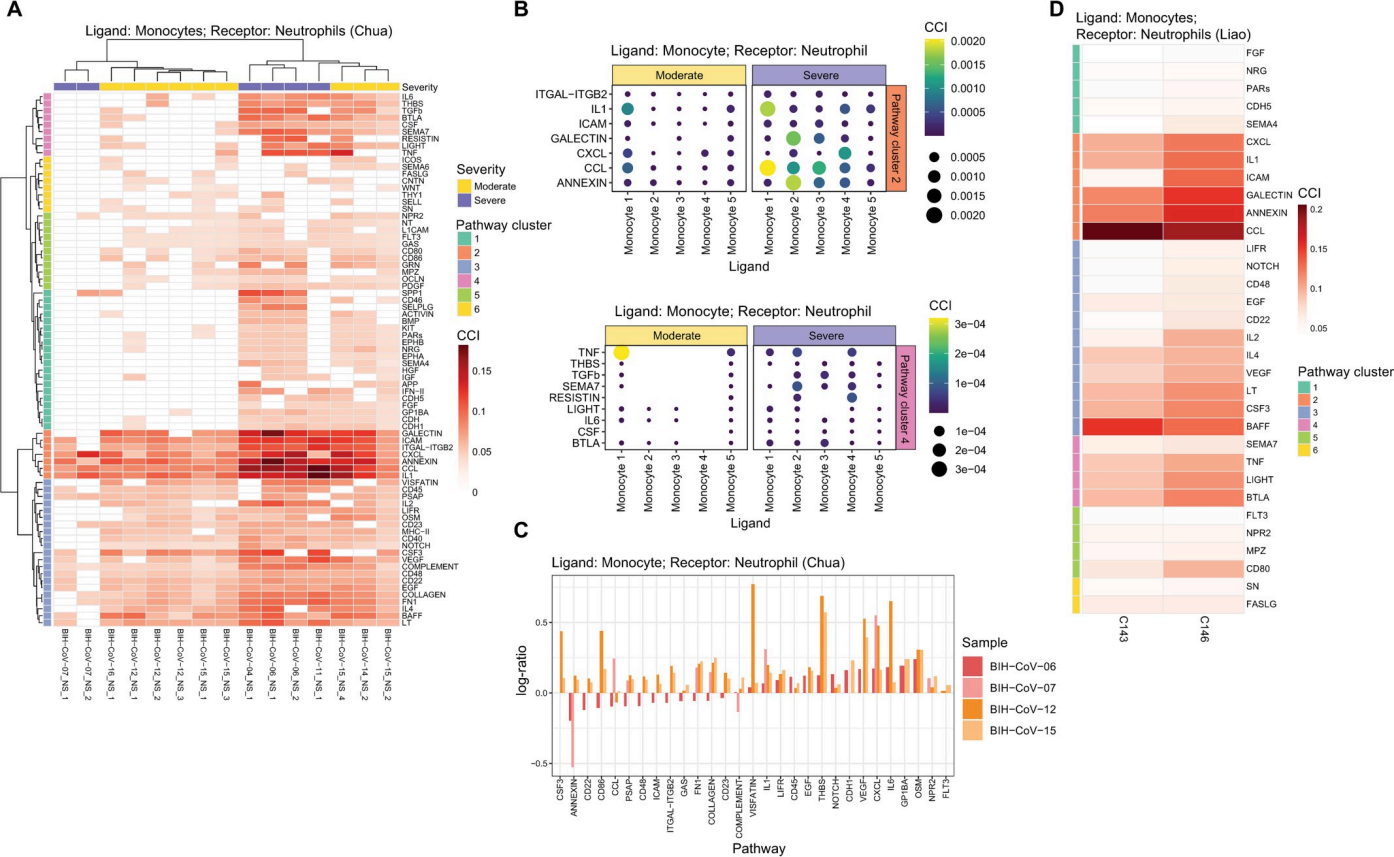
Monocyte and neutrophil interaction in COVID-19 patients. A. Heatmap of the pathway-specific cell-cell interaction (pCCI) contribution in monocytes as ligands and neutrophils as receptors in the Chua dataset, where the rows indicate the signaling pathways and columns indicate the samples. The signaling pathways are clustered into 6 groups. B. Dot plot indicating the cell-cell interaction contribution (pathway-cluster cell-cell interaction) in monocyte subgroups as ligands and neutrophils as receptors of the pathway-cluster 2 (upper panel) and pathway-cluster 4 (lower panel) as defined in (A). The columns indicate the 5 cellular subtypes of monocytes as ligands and the rows indicate the signaling pathways. A larger dot represents a higher level of cell-cell interaction. C. Bar plot indicating the log-ratio of cell-cell interaction contributions between two time points (y-axis) for longitudinal samples of 4 patients (2 moderate: BIH-CoV-12, BIH-CoV-15; 2 severe: BIH-CoV-06, BIH-CoV-07) in monocytes as ligands and neutrophils as receptors. The x-axis represents the signaling pathways. D. Heatmap of the cell-cell interaction contribution in monocytes as ligands and neutrophils as receptors for two patients (C143 and C146) in the Liao dataset that have more than 20 neutrophils, where the rows indicate the signaling pathways and columns indicate the samples. The signaling pathways are highlighted by the 6 signaling pathway clusters from (A).

Focusing on individual pathways from the Chua dataset, Fig 3A illustrates that all pathways can be broadly grouped into six large clusters. In particular, two of these pathway-clusters (pathway-cluster 2 marked by orange and pathway-cluster 4 marked by pink) are dominated by inflammatory pathways and these have significantly higher interaction between monocytes and neutrophils in severe patients compared to moderate (Fig 3A and 3B). This is consistent with findings that in the healthy immune response to SARS-CoV-2 infection, alveolar macrophages recognize and phagocytize apoptotic cells; however, under a dysfunctional immune response, excessive activation and accumulation of monocytes/macrophages and neutrophils leads to the overproduction of inflammatory cytokines which then damages the lung and other organs [39,40].

To further delineate differences between moderate and severe patients observed in Fig 2C (shown by thick red edges between monocytes and neutrophils), we investigated which subpopulations of monocytes actively interact with neutrophils (S7A and S7B Fig). The two inflammatory pathway-clusters mentioned above show that different cellular subtypes of monocytes in severe patients have significantly higher interaction scores in different pathways (Fig 3B). More specifically, we found that in severe patients, cellular subtype “monocyte 1” interacts with neutrophils through IL1, and CCL pathways, whereas interactions in moderate patients are dominated instead by TNF. S6C and S6D Fig shows that “monocyte 1” is marked by genes IL1B, IL1RN, IL8, TNFRSF1B and CCL4 and characterized by gene ontology terms “regulation of inflammatory response” as well as “regulation of apoptotic signaling pathways”. The cellular subtype “monocyte 2” (marked by highly expressed IFI27), interacts primarily with neutrophils through pathways ANNEXIN and GALECTIN, which could suggest a role for this cluster in phagocytizing dying neutrophils. The cellular subtype “monocyte 3” expressing IFIT2, IFIT3, CCL8, CXCL10, and CXCL11 shows strong signatures of type 1 interferon cell-cell signaling (Figs 3B and S6C), suggesting equal support for antiviral immunity in moderate and severe patients. Alternatively, proinflammatory signaling via CXCL interactions is mainly through cellular subtype “monocyte 4”, which highly expresses CCL2, CXCL1, CXCL2 and CXCL5 (Figs 3B and S6C).

Similar patterns are observed in the monocyte-neutrophil interaction in BALF [20] tissues where patient samples with neutrophils have higher interaction signaling from monocytes through pathways CXCL, IL1, GALECTIN, ANNEXIN, and CCL (Fig 3D) demonstrating the consistency of our cell-cell interaction results across nasopharyngeal and bronchoalveolar lavage fluid samples. The impact of CXCL and IL1 are also found among the four sets of longitudinal samples in the Chua dataset under different disease progression, suggesting an increase in interactions of signaling pathways CXCL, IL1 over time (Fig 3C). Interestingly, ANNEXIN is downregulated across sampling time since the onset of symptoms in severe patients, but is upregulated in moderate patients (Fig 3C).

### Interaction from goblet cells to immune cells are heterogeneous in moderate and severe patients

**Fig 4:**
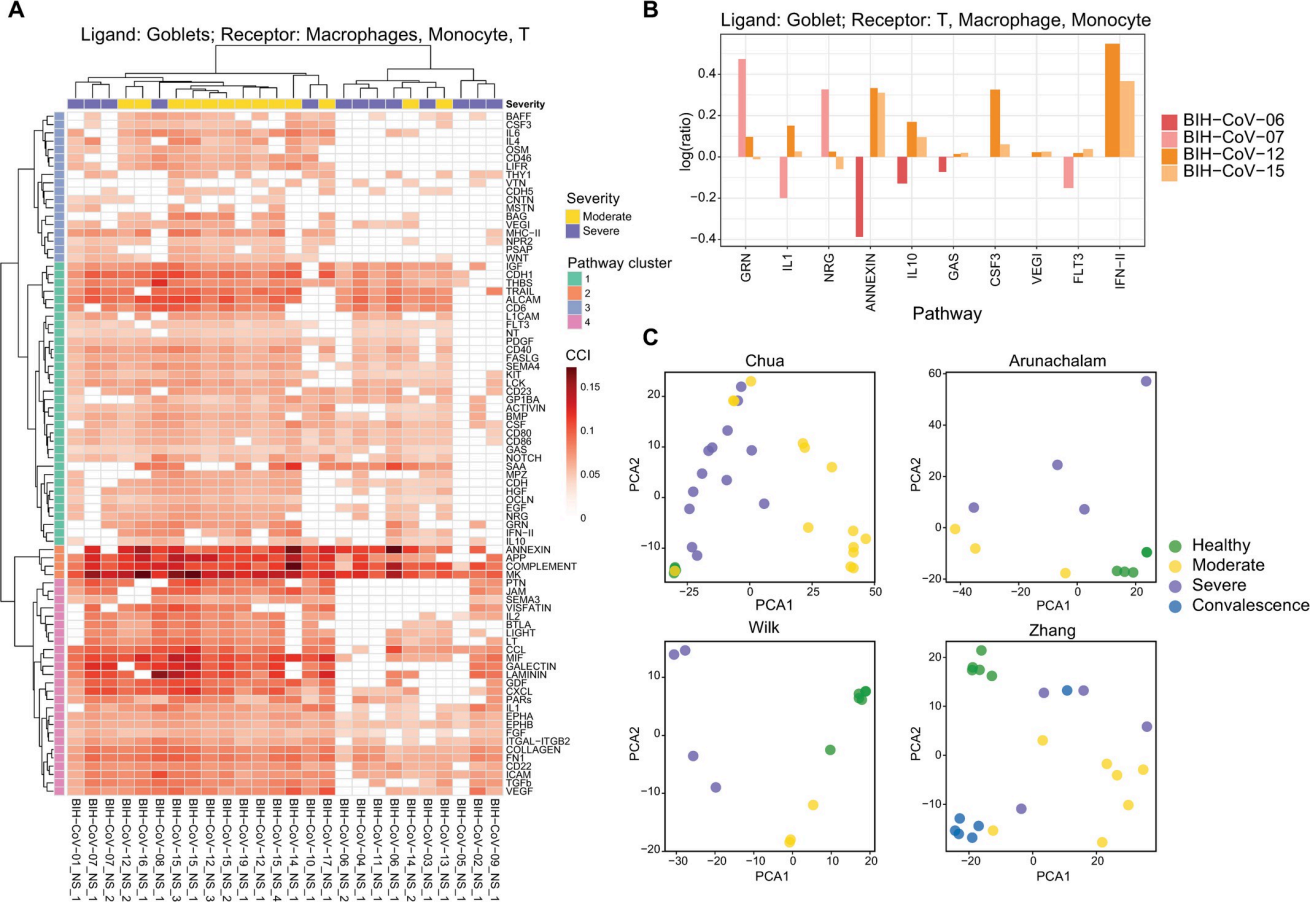
Goblet and immune cell interaction in COVID-19 patients. A. Heatmap of the pathway-specific cell-cell interaction contribution in goblets as ligands and immune cells (macrophages, monocytes and T cells) as receptors in the Chua dataset, where the rows indicate the signaling pathways and columns indicate the samples. The signaling pathways are clustered into 6 groups. B. Bar plot indicates the log-ratio of cell-cell interaction contributions between two time points (y-axis) for longitude samples of 4 patients (2 moderate: BIH-CoV-12, BIH-CoV-15; 2 severe: BIH-CoV-06, BIH-CoV-07) in goblets as ligands and immune cells (macrophages, monocytes and T cells) as receptors. The x-axis represents the signaling pathways. C. PCA for samples using the selected pathway-specific cell-cell interaction features, colored by disease severity (Healthy, Moderate, Severe, Convalescence): the Chua dataset (top left panel), the Wilk dataset (bottom left panel), the Arunachalam dataset (top right panel) and the Zhang dataset (bottom right panel) with the corresponding LOOCV accuracy rate for four datasets presented in S1 Table.

Goblet cells are found to express high levels of genes associated with innate and antiviral immune functions indicating that the nasal epithelial cells interacting with immune cells may play an important role in reducing early viral load and this is also consistent with recent literature [41]. We observe heterogeneous interaction patterns from goblet cells to immune cells across patients and pathways (Fig 4A). We observe one subgroup of severe patients (n = 3; including one deceased patient) showing clear differences in cell-cell interaction within the pathway-cluster 1 compared to moderate patients. In particular, they show a lack of interaction in the collection of pathways which includes immune signaling and costimulation pathways such as CD40, CD80/CD86, CD23 and inflammatory pathways IL6, IFN-II, and Th2 cytokines IL-4/IL10 (Fig 4A). Another subgroup of severe patients (n = 6) show clusters with a small subgroup of moderate patients that has low cell-cell interaction for antigen presentation (MHC-II), signaling pathways PTN and NPR2, and this subgroup is also lacking the Th2 cytokine IL-4 and the B cell activating factor BAFF (Fig 4A). Together, these results point to cohort heterogeneity within severe patients implicating immune co-stimulation or T cell polarizing pathways may contribute to disease severity in a particular context.

We also found differences in goblet cell interactions among COVID-19 patients at different stages of disease onset. Focusing on a specific cellular subtype of epithelial cells (goblet 5), we observe a number of increased activities in moderate patients in the ANNEXIN pathway at the late stage compared to the early stage in the moderate patients. This is most evident between cellular subtypes “goblet 5” and “monocyte 5”. This epithelial to immune cell interaction within the ANNEXIN pathway also shows an increase in patients under moderate conditions (Fig 4B). Annexin plays a role in phagocytic uptake of dying cells, can drive neutrophil detachment and apoptosis, and plays a predominant role in immune resolution [42]. Remarkably, glucocorticoids, which are effective at treating COVID-19 patients, act at least in part via upregulating Annexin I [43], suggesting that natural moderate symptoms for COVID-19 may be linked to effective endogenous immune management, or that patients that respond to glucocorticoid drugs elevate Annexin cell communication pathways that then limit further inflammation, and this response is detectable in our single-cell analysis. We have developed and provide an interactive resource (http://shiny.maths.usyd.edu.au/CovidCellInteraction/) to enable further investigation of cell-cell interaction at different resolutions from aggregated interaction between two major cell types to expression values for specific ligand-receptor pairs.

### Cell-cell interaction patterns have the potential to discriminate between moderate and severe patients

Finally, we found that information from cell-cell interactions provides a discriminating signal for patients with different disease progression. Fig 4C shows the principal components of the cell-cell interaction matrix for the Chua dataset with samples from healthy controls, moderate and severe patients highlighted. Linear discriminant analysis (LDA) shows that based on accuracy, ligand-receptor features selected from interaction from epithelial (ciliated and goblet) cells to immune cells (LOOCV = 0.8) have a higher discriminating power than using cell type proportion (LOOCV = 0.4) or ligand and receptor gene expression alone (LOOCV = 0.6). Examples of top selected pathways are THBS, BMP and EGF from pathway-cluster 1, and MHC-II and COMPLEMENT from pathway-cluster 2 (pathway-cluster defined in Fig 4A). This result is consistent regardless of the statistical machine learning methods employed (S1 Table). The accuracy rate of leave-one-out cross-validation (LOOCV) based on the first three PCs using k nearest neighbor classification (k = 3) is 84.4%, highlighting the ability of cell-cell interaction features to predict the degree of severity of patients. By repeating our workflow on the three PBMC datasets, we further demonstrate that using CCI features can achieve higher LOOCV accuracy rate than using cell type composition as features. Despite the limited samples, repeating our workflow on a smaller dataset within BALF tissues in the Liao dataset demonstrates similar findings that cell-cell communication patterns between goblet cells to immune cells has potential discriminating power.

## Discussion

A better understanding of virus and host cell interaction at the cellular level is an important component in understanding infectious disease progression and is critical for developing a treatment for the disease. In this paper, we provide a comprehensive workflow to integrate and examine multiple COVID-19 single-cell RNA-seq datasets to identify differential cell-cell interaction (CCI) pathways with respect to disease. Our results in upper airway tissues show strong intra-epithelial communication in the healthy lung, whereas the immune system then dominates communication pathways during COVID-19. We then discover that despite a higher cell-cell interaction (tCCI score) in severe patients compared to moderate patients between immune and neutrophil cells, the CCI scores between epithelial and immune cells are heterogeneous among severe patients, with a subpopulation illustrating lower CCI score when compared to moderate patients. Furthermore, features extracted from cell-cell interactions are potential signatures for discriminating between moderate and severe patients. These findings were achieved by developing a comprehensive workflow to integrate and examine multiple COVID-19 single-cell RNA-seq datasets to identify differential CCI pathways with respect to disease. Our comprehensive workflow enables scalable data integration and analysis through three important advances: (i) The “reverse” use of cell type identification to facilitate semi-supervised merging enables large scale data integration; (ii) Using multiple studies to increase the sample size to adds power to the analysis of patient single-cell data in a scalable way that enables the prediction of patient outcomes; (iii) Considering differential CCIs as features in a supervised learning framework to discriminate patient outcome.

In most multi-omics profiling in patients with COVID-19, strong acute inflammatory responses are commonly found in most of the cell types as expected. Since the airway epithelium is the primary site of infection for SARS-CoV-2 causing disease, investigating how epithelial cells interact with immune cells differentially leads to a better understanding of the initial host reaction to viral infection. Therefore, examining cell-cell communication offers an analytical approach to characterize specific cell type interaction and identify potential immune response drivers that results in different degrees of disease severity.

The importance of using a workflow that accounts for cohort heterogeneity in examining severe and moderate patients is clearly illustrated when we examine the interaction pattern between ligands in epithelial cells and various receptors in immune cells. This is different to the approach taken by Chua and colleagues [19], where a higher overall/aggregate interaction between epithelial and immune cells was identified in severe patients. Here, when we examine the cell-cell interaction relationships at the individual sample level, we observe clear cohort heterogeneity among severe patients, and furthermore, are also able to discover a subgroup of the moderate patients with higher interaction between epithelial and immune cells.

In this study, we focus on the cell communication within COVID-19 patients via ligand-receptor signaling. Several methods have been developed recently to infer such cell-cell interaction from scRNA-seq data, such as CellPhoneDB, SingleCellSignalR, NicheNet, NATMI and CellChat [7,32,33,44,45]. Most of these methods aim to identify the significant ligand and receptor gene pairs between two cell populations with the most recent method CellChat [7] that accounts for additional signaling factors. In addition, CellChat systematically categorizes the ligand-receptor pairs based on their signaling pathways, providing a comprehensive interpretation of cell-cell communication from single-cell RNA-seq. There are also other types of cell communication like physical cell interaction that can be further investigated. Technology to sequence physically interacting cells like PIC-seq has been used to investigate epithelial–immune interaction and infectious disease in mice [46]. Application of such technology in COVID-19 research will potentially allow characterization of differential physical intercellular interaction at high resolution.

Our analysis suggests the heterogeneity of cell-cell interaction patterns within patients, even if they have similar symptoms. One key variability is the sampling time since the onset of symptoms, as this may not fully capture the true underlying disease progression within each individual. Other potential factors that lead to the variability include age, gender, comorbidities and viral load. Currently, with the limited number of samples from patients with similar clinical characteristics, accounting for these uncertainties in modelling is challenging. Towards the future, as more large single-cell profiling resources in COVID-19 become publicly available, integrative analysis and meta-analysis of these studies by incorporating patient diversity to our workflow will provide a more comprehensive characterization of cell-cell interaction patterns in COVID-19 patients. Nevertheless, using the current databases our workflow supports that cell-cell interactions provide more meaningful predictions of disease progression (Fig 4C).

In summary, our novel workflow enables integrative analysis of five different COVID-19 scRNA-seq data sets with a total of 415,856 cells and 85 samples. This generalizable workflow was built on the latest single-cell analytical methods and enables the identification of differential cell-cell interaction across disease progression. We discover clear cohort heterogeneity among the severe patients in the interaction between epithelial and immune cells, with signatures that can be linked with patient outcome. Together, we provide a validated workflow for integration and analysis of diverse single-cell sequencing data to pinpoint communication networks that control disease outcome.

## Material and methods

### Computational workflow

#### Step 1—Cell type annotation

For a given dataset, we perform a cell type identification using the scClassify framework [28]. Specifically, to identify the cell types from the Chua dataset and the Liao dataset, we performed a modified version of the joint classification from scClassify that incorporates the concept of iterative supervised learning. The initial model is built from four reference datasets including annotated cell information from healthy human lungs [12,35,36]. The final cell type labels were determined by the majority vote from individual classification labels using each single reference. An additional scClassify model based on the assigned cells was then built to predict the cells that are classified as “intermediate” or “unassigned” in the previous step. To identify cell types from the PBMC datasets, we used the Wilk dataset as a reference [21] to build the model and use it to predict the cell types for the Zhang dataset and the Arunachalam dataset.

#### Step 2—Unsupervised clustering for subpopulation identification

We performed unsupervised clustering on each classified cell type to identify the cellular subtypes in the Chua dataset and the Liao dataset. For each cell type, we first calculate the deviance across cells within each sample based on a multinomial null model where each gene has a constant rate across cells. Genes with biological variations will have large deviance, indicating the null model is fitted poorly. The deviance is calculated using the function devianceFeatureSelection implemented in the R package scry (version 1.0.0) [37]. Next, we select features that are among the top 1000 largest deviances in more than 50% of the samples. We then performed negative binomial generalized principal component analysis (GLM-PCA) on the UMI matrix with the selected features (number of components is set to 30) [47]. A shared nearest neighbor graph is then built based on the GLM-PCA low-dimensional space and used as an input for Louvain clustering to identify subclusters, considering each of them as a refined cellular subtype.

To prevent over clustering, we follow a similar workflow described in clusterExperiment to collapse the identified subclusters [34]. Hierarchical clustering is first performed on the aggregated average expression of each subcluster to construct a cluster hierarchy, and then from the bottom to top, the clusters of the same branches are merged if less than 10 genes are differentially expressed (log fold change > 1, FDR < 0.01). Note that we identified some cellular subtypes (ionocytes and squamous) that are inconsistently annotated between the original Chua dataset and scClassify (classified as goblet cells). In this instance, based on marker expression, we manually reannotated these two cell types using the original annotation for the downstream analysis.

#### Step 3—Calculating cell-cell interaction (CCI)

For a given individual sample and a pair of subclusters (i.e. cellular subtypes) obtained in Step 2, we calculate the aggregated ligand-receptor interaction score based on CellChat [7]. This represents the communication probabilities among all pairs of subclusters across all ligand-receptor pairs. The CellChat algorithm aims to identify the significant ligand-receptor gene pairs between two cell populations while accounting for important signaling factors, including the expression of soluble agonists, antagonists, and stimulatory and inhibitory membrane-bound co-receptors. Finally, ligand-receptor pairs are classified into different functionally related signaling pathways. The communication probability of a signaling pathway is defined as the sum of the probabilities of its ligand-receptor pairs.

The implementation is available as R code stored at the GitHub, https://github.com/SydneyBioX/COVID_CCI_analysis and as a web shiny application at http://shiny.maths.usyd.edu.au/CovidCellInteraction/.

### Statistical formulation

The output of the cell-cell interaction analysis can be considered as a three-dimensional array representing the cell-cell interaction (CCI) score. Let *x*_*cpk*_ denote the cell-cell interaction (CCI) score generated from the computational workflow for a pair of cellular subtypes *c*, where *c*∈*C* (defined below as a set consisting of all pairs of cellular subtypes), signaling pathway *p* with *p* = 1,…,*P*, and individual sample *k* with *k* = 1,…,*K*, with *x*_*cpk*_∈[0, 1]. Each signaling pathway contains one or multiple ligand receptor pairs, curated by CellChat [7]. In general, an individual sample *k* represents the sample from one individual collected at a specific time point.

For *N* major cell types, we denote them by the sets *M*_1_, *M*_2_,…,*M*_*N*_ and within a given major cell type *M*_*i*_ consisting of *n*_*i*_ cellular subtypes $m_{i1},m_{i2},\ldots ,m_{in_{i}}$ we can write *M*_*i*_ = {*m*_*iu*_|*u* = 1,…, *n*_*i*_}. We can represent a pair of cellular subtypes as *c* = (*m*_*iu*_, *m*_*jv*_), where *i*, *j* = 1,…,*N*; *u* = 1,..,*n*_*i*_ and *v* = 1,…,*n*_*j*_. Here, we consider *m*_*iu*_ as the sender cellular subtype within major cell type *M*_*i*_ and *m*_*jv*_ as the receiver cellular subtype from major cell type *M*_*j*_. The collection of all pairs of cellular subtypes is written as *C* = {(*m*_*iu*_, *m*_*jv*_)|*i*, *j* = 1,…,*N*; *u* = 1,…,*n*_*i*_; *v* = 1,…,*n*_*j*_}. We further denote $C_{M_{i},M_{j}}$ as a subset of *C* containing only pairs of cellular subtypes from major cell type *M*_*i*_ to *M*_*j*_ which is represented as $C_{M_{i},M_{j}}=\{(m_{iu},m_{jv})|m_{iu}\in M_{i};m_{jv}\in M_{j}\}$.

For a given sample *k*, the following measures of interest are explored:

- Subtype cell-cell interaction (sCCI) between a pair of cellular subtypes *c* for an individual sample *k* is calculated as $\mathrm{sCCI}\;(c,k)=\sum_{p}x_{cpk}$. This measure totals the cell-cell interaction score across all pathways. Calculating this score for each pair of cellular subtypes and each individual sample is the same as totaling the array *X* across the pathways resulting in a |*C*|×*K* two-dimensional matrix.
- Pathway specific cell-cell interaction (pCCI) from the major cell type *M*_*i*_ to the major cell type *M*_*j*_ for a pathway *p* and an individual sample *k* is $\mathrm{pCCI}\;(M_{i},M_{j},p,k)=\sum_{c\in C_{M_{i},M_{j}}}x_{cpk}$ where $C_{M_{i},M_{j}}$ is defined as above. This is a measure that sums the cell-cell interaction scores across all cellular subtypes between any two major cell types. For each pair of (*M*_*i*_, *M*_*j*_), calculating this statistic for each pathway *p* and individual sample *k* results in a *P*×*K* matrix (see Fig 3A).
- Total CCI (tCCI) from major cell type *M*_*i*_ to major cell type *M*_*j*_ for an individual sample *k* is defined as $\mathrm{tCCI}(M_{i},M_{j},k)=\sum_{c\in C_{M_{i},M_{j}}}\sum_{p}x_{cpk}=\sum_{c\in C_{M_{i},M_{j}}}\mathrm{sCCI}(c,k),$ where $C_{M_{i},M_{j}}$ is defined as above. This is a measure that sums the cell-cell interaction scores across all cellular subtypes between two major cell types and across all pathways. For each individual sample *k*, calculating the tCCI statistic for each pair of (*M*_*i*_, *M*_*j*_) will result in a *N*×*N* matrix that can be visualized as a heatmap or network graph.
- Suppose P represents a set of pathways belonging to the same cluster termed as a pathway-cluster (see Clustering in Material and Methods). The pathway-cluster cell-cell interaction for an individual sample *k* between a pair of cellular subtypes c is defined as $\mathrm{psCCI}(c,P,k)=\frac{1}{\left|P\right|}\sum_{p\in P}x_{cpk}$.

### Association analysis for CCI

We calculate a group specific cell-cell interaction (CCI_group_) between two cellular subtypes where groups represent any treatment of interest. Here it refers to control and disease progression such as moderate and severe patients. Let $K_{\mathrm{group}}$ denote a set of individual samples under the same condition of interest, where $\left|K_{\mathrm{group}}\right|$ indicates the size of the set. For example, the total number of samples having moderate response to COVID-19 in the dataset (see Fig 2A and 2B). The CCI_group_ from the major cell types *M*_*i*_ to the major cell types *M*_*j*_ can be calculated by

${\mathrm{CCI}}_{\mathrm{group}}(M_{i},M_{j},K_{\mathrm{group}})=\frac{1}{\left|K_{\mathrm{group}}\right|}\sum_{k\in K_{\mathrm{group}}}s(\mathrm{tCCI}(M_{i},M_{j},k)),$ where $s(y)=\frac{\left(y-min(y)\right)}{\left(max(y)-min(y)\right)}$ is a scaling function to scale between individual samples. In practice, the **differential CCI** from *M*_*i*_ to *M*_*j*_ between moderate (CCI_moderate_) and severe (CCI_severe_) patients can be calculated by CCI_severe_−CCI_moderate_ measuring the differential patterns of the cell-cell interaction across different disease severity (see Fig 2C).

The pathway-cluster cell-cell interaction (used in Fig 3B) for a group of individuals *k* between a pair of cellular subtypes c is simply the sums of psCCI across individual with a group *k* and can be written as $\frac{1}{\left|K_{\mathrm{group}}||P\right|}\sum_{k\in K_{\mathrm{group}}}\sum_{p\in P}x_{cpk}=\frac{1}{\left|K_{\mathrm{group}}\right|}\sum_{k\in K_{\mathrm{group}}}\mathrm{psCCI}(c,P,k)$. For a pair of cellular subtypes *c*, calculating this statistic results in a $\left|P|\times |K_{\mathrm{group}}\right|$ matrix.

### Statistical analysis of longitudinal data

Suppose we have multiple samples collected from the same individual at different time points, say *k*_early_ and *k*_late_ then the cell-cell interaction across disease progression is the log-ratio of cell-cell interaction (illustrated in Fig 3C) between these two time points for a given pair of cell types (sender cell type *M*_*i*_, receiver cell types *M*_*j*_ within a pathway *p* is $s_{ij}=log(\mathrm{pCCI}(M_{i,}M_{j},k_{\mathrm{late}})/(\mathrm{pCCI}(M_{i,}M_{j},k_{\mathrm{early}})+c))$, where *c* is a constant to regularize the ratio in the case when pCCI(*M*_*i*_, *M*_*j*_, *k*_early_) equal to zero. We set *c* = 0.1*d*, where *d* is the smallest non-zero value of pCCI(*M*_*i*_, *M*_*j*_, *k*_early_). For pCCI(*M*_*i*_, *M*_*j*_, *k*_late_) = 0, *s*_*ij*_ is set to zero. A positive value of *s*_*ij*_ indicates the pathway is upregulated at the late stage, while a negative value indicates the pathway is downregulated.

### Clustering

We group various pathways based on the similarity of intercellular communication patterns using hierarchical clustering with Euclidean distance and ward.D2 agglomerative method implemented in the function hclust in R.

### Data and preprocessing

[A] Chua dataset—The raw count matrix and metadata containing patient information are downloaded from FigShare: https://doi.org/10.6084/m9.figshare.12436517 [19]. This dataset includes 19 patients with critical or moderate disease as well as 5 healthy controls.

[B] Liao dataset—The raw count matrices of single-cell RNA-seq data from bronchoalveolar lavage fluid was downloaded from the National Center for Biotechnology Information (NCBI) Gene Expression Omnibus (GEO) under the accession number GSE145926. This dataset has 3 healthy controls, 3 moderate patients and 6 severe patients [20].

[C] Wilk dataset—The raw count matrices of single-cell RNA-seq data from PBMC with metadata were downloaded from the COVID-19 Cell Atlas: https://www.covid19cellatlas.org/#wilk20 [21]. This dataset contains 6 healthy controls, 3 moderate patients and 4 severe patients.

[D] Arunachalam dataset—The raw count matrices of single-cell RNA-seq data from PBMC and the clinical information were downloaded from GEO under accession number GSE155673. This dataset has 5 healthy controls, 3 moderate patients and 4 severe patients [23]. The cells with more than 20% mitochondrial proportion and UMI count greater than 50,000 are removed from the downstream analysis.

[E] Zhang dataset—The raw sequence files of single-cell RNA-seq data from PBMC are downloaded from the Genome Sequence Archive of the Beijing Institute of Genomics (BIG) Data Center, BIG, Chinese Academy of Science using the accession code HRA000150 [22]. Cell Ranger (v3.0.2) with human reference version GRCh38 were used to generate the raw count matrices. The dataset includes 5 healthy controls, 7 moderate patients and 4 severe patients. Only the cells retained from the original study are used.

Processing: For each dataset, we performed size factor standardization and log transformation on the raw count expression matrices using the logNormCount function in the R package scater (version 1.16.2) and generated log transformed gene expression matrices for analysis.

### PBMC data integration

We integrated the six PBMC datasets using a modified version of scMerge [37]. Here, cell types annotated by scClassify are used as an input to scMerge to construct pseudo-bulk expression profiles. The resulting profiles are used to identify mutual nearest subgroups as pseudo-replicates and to estimate parameters of the scMerge model.

### Machine learning for discrimination

To select the cell-cell interaction features that discriminate across samples under different conditions, we performed a Kruskal-Wallis rank sum test on pathway-specific cell-cell interaction (pCCI) to select the pathways that are significantly different across samples from healthy controls, moderate patients and severe patients. Feature selection is based on pCCI features with an adjusted p-value less than 0.1 for the Chua dataset, less than 0.2 for the Wilk and Zhang datasets and less than 0.4 for the Arunachalam dataset, we termed these selected features as “Top CCI”. For the Chua dataset, we also selected the top pCCI from the cell-cell interaction between the two major epithelial cell types (Goblet and Ciliated) and the immune cell types (B cells, dendritic cells, macrophages, monocytes and T cells), termed as “Epi-Immune CCI”. We further considered cell type proportion as another type of feature. The classification model to predict the samples’ condition is built with linear discriminant analysis (LDA) and random forest (RF) on the selected features (Top CCI, Epi-Immune CCI, and cell type proportion) as well as k nearest neighbor classification (with k = 1, 3) using the first 3 principal components of the pCCI matrix. The classification performance was determined by leave-one-out cross-validation.

### Gene ontology analysis

Differential gene expressions were identified using moderated t-statistics implemented in the R package limma (version 3.44.3). The gene set over-representation analysis for the significant DE genes (top 100 genes selected) with biological process (BP) gene ontology is measured using the “enrichGO” function in the R package clusterProfiler (version 3.16.0) [48]. Significant GO terms are defined by q-value < 0.1.

### Interactive graphics implementation

To facilitate the interpretation of the complex data set, we have created an online interactive tool which allows researchers to explore different parts of the data. The first tab of the tool contains four columns. The first column allows the user to select two groups (or individual samples) to compare and it displays the associated cell-cell interaction network. The second column shows the difference between the two selected groups (or samples) in a heatmap and network form. Selecting a cell type pair from the heatmap dissects the interaction into individual pathways and sub-cell types, displayed in the third column. Selecting a pathway on this heatmap further dissects the activity into individual ligand-receptor pairs, displayed in the fourth column. The second tab of the tool allows the user to select a gene and its mean expression is shown for each cell type and sample. The user can also select a ligand cell type and a receptor cell type and the activity of all pathways between these cell types and involving the selected gene are shown.

## Supporting information

S1 Fig (A) tSNE plots with the Chua dataset, colored by the disease condition (left panel), and individual sample (right panel). (B) Cell type composition of each individual sample in the Chua dataset. (C) Boxplots of marker expression for each reannotated cell type.
(DOCX)

S2 Fig (A) tSNE plot of scRNA-seq data from BALF (the Liao dataset), colored by the reannotation from scClassify. (B) Cell type composition of each sample in the Liao dataset. (C) Heatmap indicating the difference of group specific cell-cell interaction between different cell types in severe patients and moderate patients in the Liao dataset. Red color indicates a higher interaction in severe patients and blue color indicates a higher interaction in moderate patients. Rows indicate the sender cell types and columns indicate the receiver cell types.
(DOCX)

S3 Fig Comparison of cell-cell interactions in COVID-19 patients of varying severities. (A-C) Network representing the group specific cell-cell interaction (CCI_group_) considering different disease severity as groups in the Chua dataset from (A) healthy controls (B) moderate patients and (C) severe patients. The nodes represent major cell types and the edges represent aggregate tCCI interaction signals across individuals from the same group. Thicker edges indicate stronger cell-cell interaction signals. (D) Network representing the difference of cell-cell interaction between severe and moderate patients. The nodes represent cell types and an edge measures the difference in cell-cell interaction. A red edge indicates an interaction higher in severe patients and a blue edge indicates an interaction higher in moderate patients.
(DOCX)

S4 Fig (A-C) Heatmaps indicating the group specific cell-cell interaction between different cell types in (A) healthy controls (B) moderate patients (C) severe patients for the Chua dataset. Rows indicate the sender cell types and columns indicate the receiver cell types. (D-F) Heatmaps indicate the difference in group specific cell-cell interaction between different cell types in (D) severe patients and healthy controls (E) moderate patients and healthy controls (F) severe patients and moderate patients for the Chua dataset. Red color indicates a higher interaction in severe patients and blue color indicates a higher interaction in moderate patients. Rows indicate the sender cell types and columns indicate the receiver cell types.
(DOCX)

S5 Fig Cell type composition of each individual sample in the six PBMC datasets. (DOCX)

S6 Fig (A) Heatmaps indicating the group specific cell-cell interaction between different cell types in healthy controls (left panel), moderate patients (middle panel) and severe patients (right panel) for the PBMC dataset collection. Rows indicate the sender cell types and columns indicate the receiver cell types. (B) Heatmaps indicate the difference in group specific cell-cell interaction between different cell types in moderate patients and healthy controls (left panel), severe patients and healthy controls (middle panel) and severe patients and moderate patients (right panel) for the PBMC dataset collection. Red color indicates a higher interaction in severe patients and blue color indicates a higher interaction in moderate patients. Rows indicate the sender cell types and columns indicate the receiver cell types.
(DOCX)

S7 Fig (A) tSNE plot of monocytes in the Chua dataset, colored by the five cellular subtypes of monocytes. (B) Stacked bar plots representing the number of cells for healthy, moderate and severe groups. The x-axis represents the five cellular subtypes of monocytes for the Chua dataset. (C) Heatmap indicates the scaled average marker expression of the five cellular subtypes of monocytes. (D) Gene ontology analysis for the cellular subtypes of monocytes.
(DOCX)

S1 Table LOOCV accuracy rate for four datasets using four classification methods: KNN (K = 1), KNN (K = 3), linear discriminant analysis (LDA), and random forest (RF). The row “Top CCI” refers to classification results based on features selected by Kruskal-Wallis rank sum test on pathway-specific cell-cell interaction (pCCI) (See Material and Methods section for more details). The row “Epi-Immune CCI” refers to classification results based on features selected from the cell-cell interaction between the two major epithelial cell types (Goblet and Ciliated) and the immune cell types (B cells, dendritic cells, macrophages, monocytes and T cells). The row “cell type proportion” refers to classification results based on the cell type proportion. The highlighted cells indicated the best performing signature(s) for each of the classification methods.
(DOCX)
