# Supplementary material for: Scalable workflow for characterization of cell-cell communication in COVID-19 patients
Source: PLoS Comput Biol. 2022 Oct 5;18(10):e1010495. doi: 10.1371/journal.pcbi.1010495 (PMC9534414; doi:10.1371/journal.pcbi.1010495)
Supplement: S1 Fig — (A) tSNE plots with the Chua dataset, colored by the disease condition (left panel), and individual sample (right panel). (B) Cell type composition of each individual sample in the Chua dataset. (C) Boxplots of marker expression for each reannotated cell type. (DOCX) [file pcbi.1010495.s001.docx]

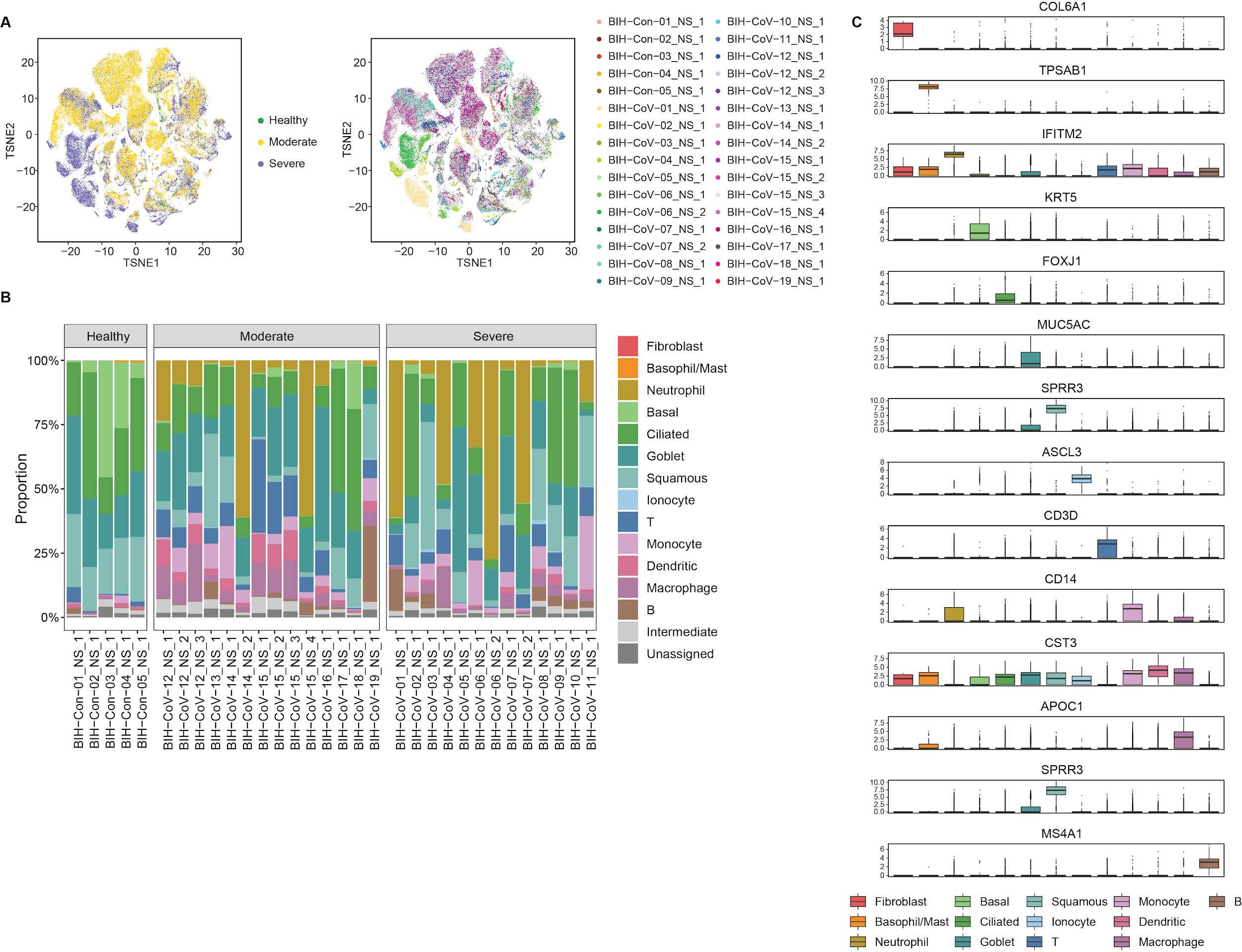


**S1 Fig** (A) tSNE plots with the Chua dataset, colored by the disease condition (left panel), and individual sample (right panel). (B) Cell type composition of each individual sample in the Chua dataset. (C) Boxplots of marker expression for each reannotated cell type.
