# Supplementary material for: Scalable workflow for characterization of cell-cell communication in COVID-19 patients
Source: PLoS Comput Biol. 2022 Oct 5;18(10):e1010495. doi: 10.1371/journal.pcbi.1010495 (PMC9534414; doi:10.1371/journal.pcbi.1010495)
Supplement: S2 Fig — (A) tSNE plot of scRNA-seq data from BALF (the Liao dataset), colored by the reannotation from scClassify. (B) Cell type composition of each sample in the Liao dataset. (C) Heatmap indicating the difference of group specific cell-cell interaction between different cell types in severe patients and moderate patients in the Liao dataset. Red color indicates a higher interaction in severe patients and blue color indicates a higher interaction in moderate patients. Rows indicate the sender cell types and columns indicate the receiver cell types. (DOCX) [file pcbi.1010495.s002.docx]

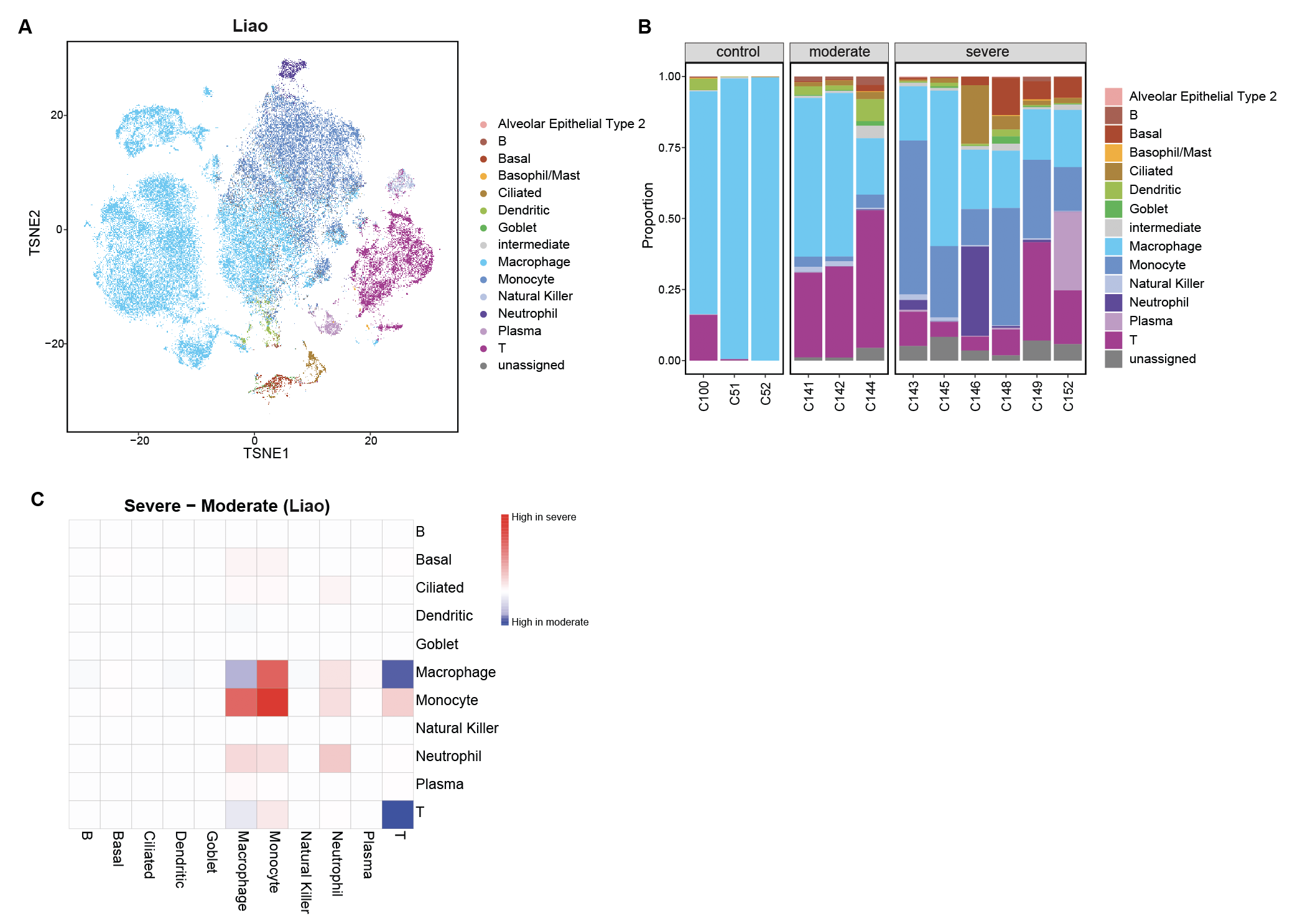


**S2 Fig** (A) tSNE plot of scRNA-seq data from BALF (the Liao dataset), colored by the reannotation from scClassify. (B) Cell type composition of each sample in the Liao dataset. (C) Heatmap indicating the difference of group specific cell-cell interaction between different cell types in severe patients and moderate patients in the Liao dataset. Red color indicates a higher interaction in severe patients and blue color indicates a higher interaction in moderate patients. Rows indicate the sender cell types and columns indicate the receiver cell types.
