# Supplementary material for: Scalable workflow for characterization of cell-cell communication in COVID-19 patients
Source: PLoS Comput Biol. 2022 Oct 5;18(10):e1010495. doi: 10.1371/journal.pcbi.1010495 (PMC9534414; doi:10.1371/journal.pcbi.1010495)
Supplement: S3 Fig — (A-C) Network representing the group specific cell-cell interaction (CCIgroup) considering different disease severity as groups in the Chua dataset from (A) healthy controls (B) moderate patients and (C) severe patients. The nodes represent major cell types and the edges represent aggregate tCCI interaction signals across individuals from the same group. Thicker edges indicate stronger cell-cell interaction signals. (D) Network representing the difference of cell-cell interaction between severe and moderate patients. The nodes represent cell types and an edge measures the difference in cell-cell interaction. A red edge indicates an interaction higher in severe patients and a blue edge indicates an interaction higher in moderate patients. (DOCX) [file pcbi.1010495.s003.docx]

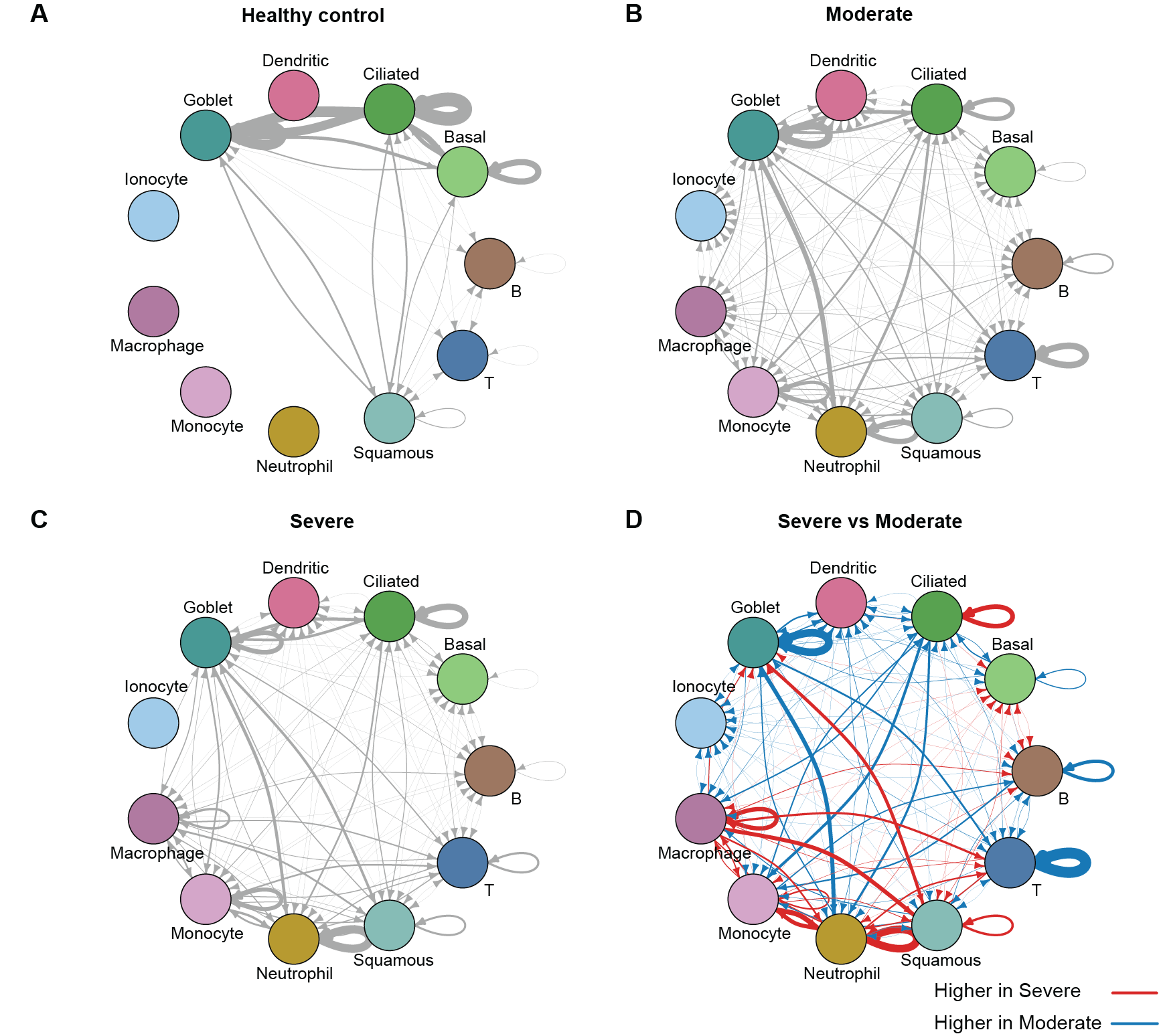


**S3 Fig: Comparison of cell-cell interactions in COVID-19 patients of varying severities.** (A-C) Network representing the group specific cell-cell interaction ($\text{CCI}_{\text{group}}$) considering different disease severity as groups in the Chua dataset from (A) healthy controls (B) moderate patients and (C) severe patients. The nodes represent major cell types and the edges represent aggregate tCCI interaction signals across individuals from the same group. Thicker edges indicate stronger cell-cell interaction signals. (D) Network representing the difference of cell-cell interaction between severe and moderate patients. The nodes represent cell types and an edge measures the difference in cell-cell interaction. A red edge indicates an interaction higher in severe patients and a blue edge indicates an interaction higher in moderate patients.
