# Supplementary material for: Scalable workflow for characterization of cell-cell communication in COVID-19 patients
Source: PLoS Comput Biol. 2022 Oct 5;18(10):e1010495. doi: 10.1371/journal.pcbi.1010495 (PMC9534414; doi:10.1371/journal.pcbi.1010495)
Supplement: S4 Fig — (A-C) Heatmaps indicating the group specific cell-cell interaction between different cell types in (A) healthy controls (B) moderate patients (C) severe patients for the Chua dataset. Rows indicate the sender cell types and columns indicate the receiver cell types. (D-F) Heatmaps indicate the difference in group specific cell-cell interaction between different cell types in (D) severe patients and healthy controls (E) moderate patients and healthy controls (F) severe patients and moderate patients for the Chua dataset. Red color indicates a higher interaction in severe patients and blue color indicates a higher interaction in moderate patients. Rows indicate the sender cell types and columns indicate the receiver cell types. (DOCX) [file pcbi.1010495.s004.docx]

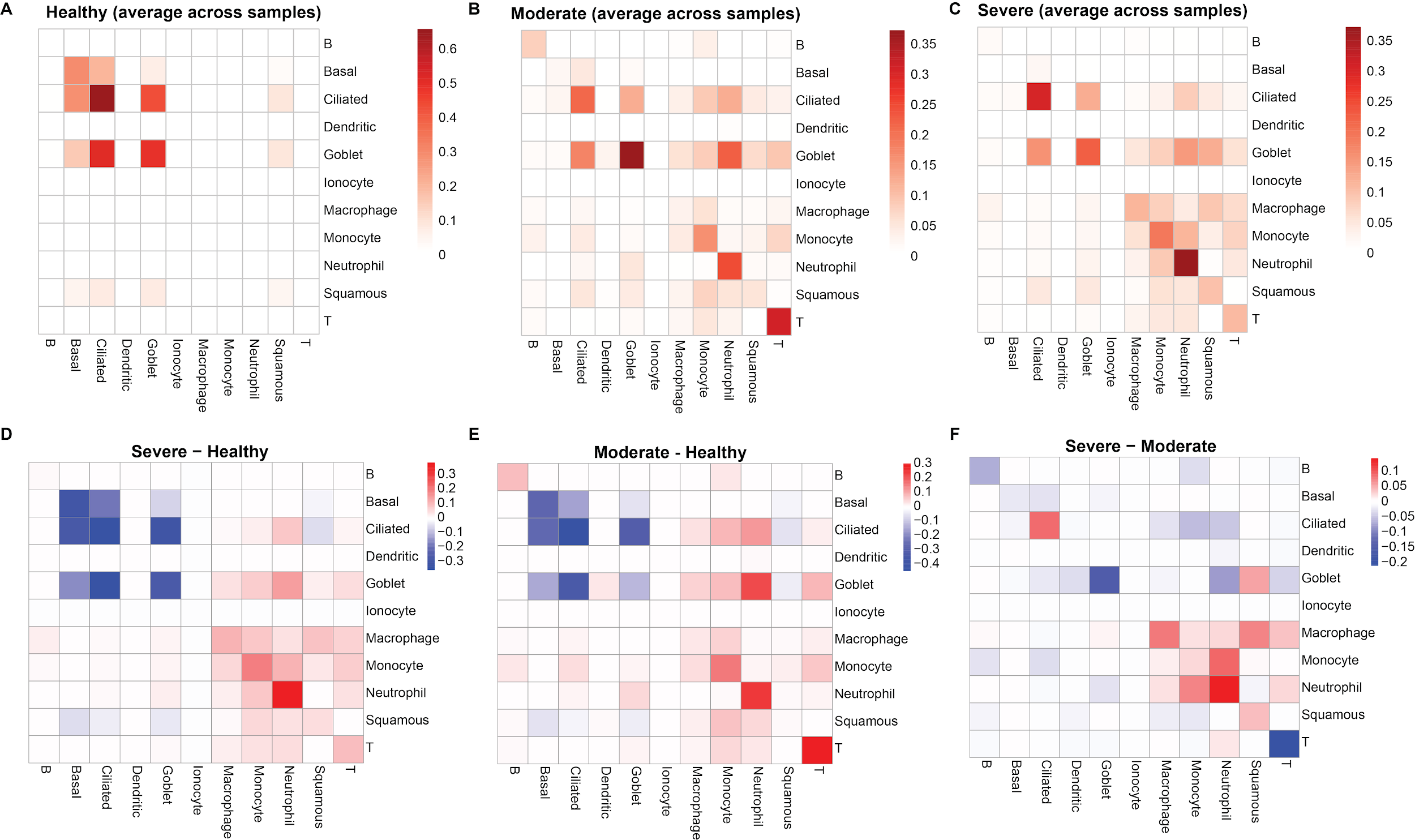


**S4 Fig** (A-C) Heatmaps indicating the group specific cell-cell interaction between different cell types in (A) healthy controls (B) moderate patients (C) severe patients for the Chua dataset. Rows indicate the sender cell types and columns indicate the receiver cell types. (D-F) Heatmaps indicate the difference in group specific cell-cell interaction between different cell types in (D) severe patients and healthy controls (E) moderate patients and healthy controls (F) severe patients and moderate patients for the Chua dataset. Red color indicates a higher interaction in severe patients and blue color indicates a higher interaction in moderate patients. Rows indicate the sender cell types and columns indicate the receiver cell types.
