# Supplementary material for: Scalable workflow for characterization of cell-cell communication in COVID-19 patients
Source: PLoS Comput Biol. 2022 Oct 5;18(10):e1010495. doi: 10.1371/journal.pcbi.1010495 (PMC9534414; doi:10.1371/journal.pcbi.1010495)
Supplement: S5 Fig — (DOCX) [file pcbi.1010495.s005.docx]

**S5 Fig.** Cell type composition of each individual sample in the six PBMC datasets.
