# Supplementary material for: Scalable workflow for characterization of cell-cell communication in COVID-19 patients
Source: PLoS Comput Biol. 2022 Oct 5;18(10):e1010495. doi: 10.1371/journal.pcbi.1010495 (PMC9534414; doi:10.1371/journal.pcbi.1010495)
Supplement: S6 Fig — (A) Heatmaps indicating the group specific cell-cell interaction between different cell types in healthy controls (left panel), moderate patients (middle panel) and severe patients (right panel) for the PBMC dataset collection. Rows indicate the sender cell types and columns indicate the receiver cell types. (B) Heatmaps indicate the difference in group specific cell-cell interaction between different cell types in moderate patients and healthy controls (left panel), severe patients and healthy controls (middle panel) and severe patients and moderate patients (right panel) for the PBMC dataset collection. Red color indicates a higher interaction in severe patients and blue color indicates a higher interaction in moderate patients. Rows indicate the sender cell types and columns indicate the receiver cell types. (DOCX) [file pcbi.1010495.s006.docx]

**S6 Fig** (A) Heatmaps indicating the group specific cell-cell interaction between different cell types in healthy controls (left panel), moderate patients (middle panel) and severe patients (right panel) for the PBMC dataset collection. Rows indicate the sender cell types and columns indicate the receiver cell types. (B) Heatmaps indicate the difference in group specific cell-cell interaction between different cell types in moderate patients and healthy controls (left panel), severe patients and healthy controls (middle panel) and severe patients and moderate patients (right panel) for the PBMC dataset collection. Red color indicates a higher interaction in severe patients and blue color indicates a higher interaction in moderate patients. Rows indicate the sender cell types and columns indicate the receiver cell types.
