# Supplementary material for: Scalable workflow for characterization of cell-cell communication in COVID-19 patients
Source: PLoS Comput Biol. 2022 Oct 5;18(10):e1010495. doi: 10.1371/journal.pcbi.1010495 (PMC9534414; doi:10.1371/journal.pcbi.1010495)
Supplement: S7 Fig — (A) tSNE plot of monocytes in the Chua dataset, colored by the five cellular subtypes of monocytes. (B) Stacked bar plots representing the number of cells for healthy, moderate and severe groups. The x-axis represents the five cellular subtypes of monocytes for the Chua dataset. (C) Heatmap indicates the scaled average marker expression of the five cellular subtypes of monocytes. (D) Gene ontology analysis for the cellular subtypes of monocytes. (DOCX) [file pcbi.1010495.s007.docx]

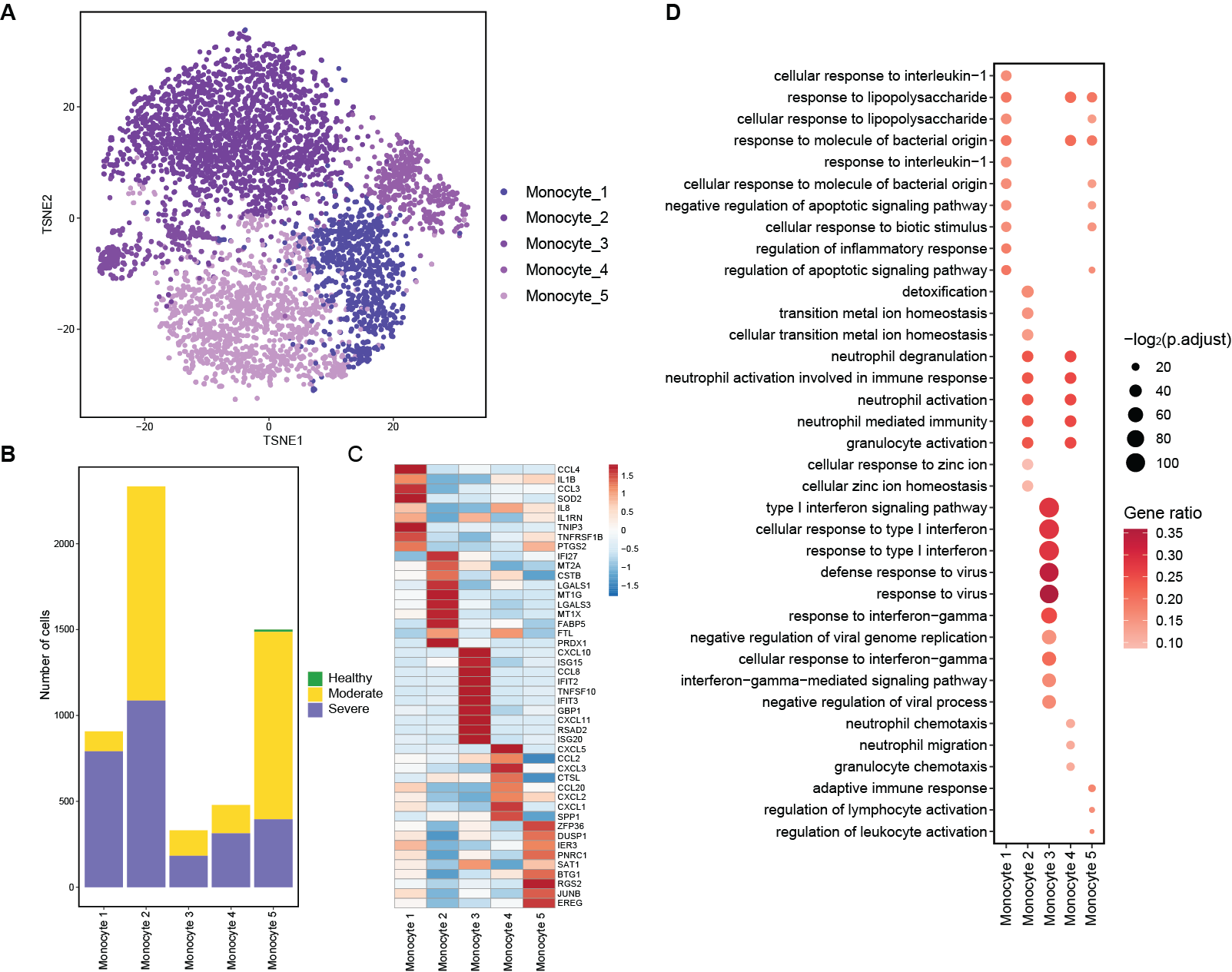


**S7 Fig** (A) tSNE plot of monocytes in the Chua dataset, colored by the five cellular subtypes of monocytes. (B) Stacked bar plots representing the number of cells for healthy, moderate and severe groups. The x-axis represents the five cellular subtypes of monocytes for the Chua dataset. (C) Heatmap indicates the scaled average marker expression of the five cellular subtypes of monocytes. (D) Gene ontology analysis for the cellular subtypes of monocytes.
