# Supplementary material for: Scalable workflow for characterization of cell-cell communication in COVID-19 patients
Source: PLoS Comput Biol. 2022 Oct 5;18(10):e1010495. doi: 10.1371/journal.pcbi.1010495 (PMC9534414; doi:10.1371/journal.pcbi.1010495)
Supplement: S1 Table — The row “Top CCI” refers to classification results based on features selected by Kruskal-Wallis rank sum test on pathway-specific cell-cell interaction (pCCI) (See Material and Methods section for more details). The row “Epi-Immune CCI” refers to classification results based on features selected from the cell-cell interaction between the two major epithelial cell types (Goblet and Ciliated) and the immune cell types (B cells, dendritic cells, macrophages, monocytes and T cells). The row “cell type proportion” refers to classification results based on the cell type proportion. The highlighted cells indicated the best performing signature(s) for each of the classification methods. (DOCX) [file pcbi.1010495.s008.docx]

**S1 Table**

LOOCV accuracy rate for four datasets using four classification methods: KNN (K = 1), KNN (K = 3), linear discriminant analysis (LDA), and random forest (RF). The row “Top CCI” refers to classification results based on features selected by Kruskal-Wallis rank sum test on pathway-specific cell-cell interaction (pCCI) (See Material and Methods section for more details). The row “Epi-Immune CCI” refers to classification results based on features selected from the cell-cell interaction between the two major epithelial cell types (Goblet and Ciliated) and the immune cell types (B cells, dendritic cells, macrophages, monocytes and T cells). The row “cell type proportion” refers to classification results based on the cell type proportion. The highlighted cells indicated the best performing signature(s) for each of the classification methods.

Chua

|  | KNN (K = 1) | KNN (K = 3) | LDA | RF |
| --- | --- | --- | --- | --- |
| Top CCI | 0.75 | 0.72 | 0.62 | 0.66 |
| Epi-Immune CCI | 0.81 | 0.72 | 0.78 | 0.72 |
| Cell type proportion | 0.47 | 0.5 | 0.44 | 0.56 |

Arunachalam

|  | KNN (K = 1) | KNN (K = 3) | LDA | RF |
| --- | --- | --- | --- | --- |
| Top CCI | 0.83 | 0.67 | 0.50 | 0.67 |
| Cell type proportion | 0.67 | 0.42 | 0.67 | 0.75 |

Wilk

|  | KNN (K = 1) | KNN (K = 3) | LDA | RF |
| --- | --- | --- | --- | --- |
| Top CCI | 0.92 | 0.92 | 1.00 | 0.77 |
| Cell type proportion | 0.46 | 0.46 | 0.62 | 0.69 |

Zhang

|  | KNN (K = 1) | KNN (K = 3) | LDA | RF |
| --- | --- | --- | --- | --- |
| Top CCI | 0.68 | 0.73 | 0.82 | 0.82 |
| Cell type proportion | 0.36 | 0.27 | 0.64 | 0.68 |
